# Comparison of presentation and disease progression between gene positive and gene negative patients evaluated for arrhythmogenic right ventricular cardiomyopathy

**DOI:** 10.1186/1532-429X-17-S1-P334

**Published:** 2015-02-03

**Authors:** Paweena Chungsomprasong, Robert Hamilton, Meena Fatah, Yousef Etoom, Sindu Govindapillai, Cedric Manlhiot, Shi-Joon Yoo, Brian W McCrindle, Lars Grosse-Wortmann

**Affiliations:** 1 The Hospital for Sick Children, Toronto, ON, Canada

## Background

Arrhythmogenic right ventricular cardiomyopathy (ARVC) is a progressive disease. However, the likelihood and rate of progression are unknown, especially in children and adolescents. Therefore, the optimal timing of serial investigations with cardiac magnetic resonance (CMR) and other tests in this age group is uncertain. Further, it is unknown whether gene positive patients differ from gene negative patients in their presentation and disease progression. The aim of this study is to determine the presentation and disease progression of patients referred for an ARVC work-up, dependent on their genetic results.

## Methods

For this retrospective study serial data in patients with a known pathogenic mutation for ARVC (gene positive group) were compared to those in gene-negative patients. The diagnosis was made according to the revised Task Force criteria (TFC). CMR, ECG, and signal average ECG results as well as clinical findings were recorded during each (serial) work-up.

## Results

Twenty-one gene positive and 102 gene negative were identified. The patients were followed for a period of 4.3±3.1 years (0-13.9). The age at initial presentation was similar in both groups (10.5 ± 4.3 and 11.7 ± 5.9 years, respectively). A higher percentage of gene positive patients (52.4% vs. 17.6% in the gene negative group, p=0.004) were referred for family history. Fewer patients in the gene positive group (33.0% vs. 72.8%, p=0.001) were symptomatic at presentation. In both groups, the number of patients who progressed from asymptomatic to symptomatic was very low (4.7% and 1% in the gene negative and positive groups, respectively, p=0.57). The rate of progression of ventricular volumes and ejection fractions of both ventricles and of ECG findings did not differ significantly between the two groups.

## Conclusions

Gene negative and positive patients are referred at similar ages. Our results indicate that most gene negative patients are referred for symptoms while most gene positive patients undergo a work-up because of a positive family history. Patients who are asymptomatic at presentation are unlikely to develop symptoms during childhood.

While important for the diagnosis according to the TFC, an identification of an ARVC gene does not appear to confer a higher risk of disease progression in childhood.

## Funding

N/A.

